# Work–Family Conflict on Children’s Internet Addiction: Role of Parenting Styles in Korean Working Mother

**DOI:** 10.3390/ijerph18115774

**Published:** 2021-05-27

**Authors:** Hwa-Mi Yang, Hye-Ryoung Kim

**Affiliations:** 1 College of Nursing, Daejin University, Pocheon-si 11159, Korea; yhm2021@daejin.ac.kr; 2 College of Nursing, Shinhan University, Dongducheon 11340, Korea

**Keywords:** conflict, parenting, addictive behavior, children, family

## Abstract

Based on spillover and crossover models in the family system, we hypothesized the mediating effect of parenting style in the association between maternal work–family conflict (WFC) and children’s problematic internet (PIU). This is a cross-sectional study using data from the 10th wave Panel Study on Korean Children (PSKC) in 2017. The study subjects were 707 mothers and their children. The WFC was measured using the Marshall and Barnett scale, parenting style by the Parenting Styles and Dimension Questionnaire developed by Robinson, and the PIU of a child by the K-Scale for adolescent observers. As a result, maternal WFC had a positive association with the PIU of a child. Maternal WFC also had a link with parenting styles. Specifically, WFC had a negative association with an authoritative parenting style, and a positive association with authoritarian and permissive parenting styles. Regarding the relationship between maternal WFC and the PIU of a child, parenting styles showed a mediating effect for authoritative (z = 2.08, *p* = 0.037), authoritarian (z = 2.71, *p* = 0.007), and permissive (z = 3.14, *p* = 0.002). Based on the results, we assert that when planning an intervention to reduce children’s PIU for working mothers, a multifaceted approach is essential, including both WFC and parenting behavior.

## 1. Introduction

### 1.1. Children’s Problematic Internet Use

Internet use has become an indispensable part of modern life, and, as a result, problematic internet use (PIU) is becoming a public health concern. In particular, excessive internet use at a young age is known to cause psychological, physical, and behavioral problems, such as attention disorder, cognitive impairment, and obesity related to a lack of physical activity [1,2,3].

PIU is expressed in various terms such as “internet addiction”, “smartphone addiction”, and “digital addiction”, yet there is no consensus on the definition. However, it is characterized by excessive internet use beyond what is intended, is uncontrollable, and causes adjustment problems in daily life through dependent behavior and an obsession with internet use [4]. The prevalence of PIU is reported in 6.3% to 16% of children and adolescents [5].

A study of elementary school students in Korea found that PIU is related to gender, a mother’s educational level, spending time without parents, media exposure time, personal feelings such as happiness, and parenting style [6]. Influencing factors related to PIU of children and adolescents are classified into family, school, and individual dimensions. Although factors such as parenting style, peer relationship, and self-esteem have been studied, it is still scarce and requires further study [7].

The family environment is critical in developing children’s healthy lifestyles, and parenting styles from parent–child relationships might influence the PIU of children. Parents of adolescents with PIU showed poor parenting more frequently [8]. According to the family system theory, the family is the sum of relationships and behaviors, and parents and children influence each other in the parent–child relationship, the subsystem of the family [9]. The mechanism by which parents and children influence each other can be explained by the crossover model. The crossover model was introduced by Bolger, DeLongis, Kessler, and Wethington [10]. Crossover refers to the interpersonal process that facilitates an emotional or behavioral transfer between people. An example of crossover is when work stress or psychological strain experienced by parents affects the level of the strain on a child in the family system [9]. On the other hand, spillover is an intrapersonal process that facilitates stress transfer across domains. An example of spillover is work–family conflicts.

### 1.2. Maternal Work–Family Conflict

Work–family conflict (WFC) is an inter-role strain, arising from the two life domains, work and family, and it means that tension and time pressure at work affect family life [11]. Mothers who have enrolled in the labor market are particularly known to be under considerable stress to meet the needs of their work and family [12]. In Korea, married women still assume the duty of care through traditional gender roles. It appears that most working mothers also play a significant role in family care and housework at home, and face work–family conflict [13]. Working mothers in Korea had the lowest level of support from spouses and employees, lower than in Israel or the United States, and such factors are related to high levels of work–family conflict [14].

Specifically, maternal WFC has been reported to be closely related to children’s problematic behaviors [15]. Maternal WFC as strain-based factors might closely relate to the PIU of a child. However, despite the underlying focus of WFC on family health and well-being, we have limited knowledge about the impact of role-based stressors, such as WFC, on child health [16].

### 1.3. The Role of Parenting Styles

Parenting style is closely related to levels of stress in the family system. WFC can be an essential factor in determining parenting behavior [17]. A higher WFC level was associated with less parental warmth, inconsistency, and poor parent–child relationships [15,18]. Dianne Baumrind classified parent–child relationships as authoritative, authoritarian, or permissive parenting style [19]. The authoritative parenting style is characterized by relatively reasonable control in the context of emotions and a high level of responsiveness and consistency in discipline. In contrast, the authoritarian parenting style is characterized by a low level of warmth/responsiveness and strict discipline. Korean working mothers tend to adopt a rigorous parenting style, showing strict control over their children. They made most decisions about their children’s activities, leaving little room for their opinions, more so than Korean American working mothers [20]. Permissive parenting style is characterized by parenting behavior with little or no rules or restrictions on the child, and low levels of control [19,21]. A study reported that a higher level of WFC was associated with the permissive or authoritative parenting style, and a lower level of WFC was associated with the authoritative parenting style [21]. 

A child who engages in PIU tends to communicate less with their parents. The PIU of a child might be a refuge from strict parenting pressures [22]. It was reported that the majority of children with PIU had experienced parenting behaviors such as excessive punishment and under-responsiveness [23]. The authoritarian or permissive styles of parents were a high risk for the child’s PIU [24,25]. Maternal parenting behaviors could influence PIU of the child [26].

### 1.4. Research Purpose and Hypothesis

**Figure 1:**
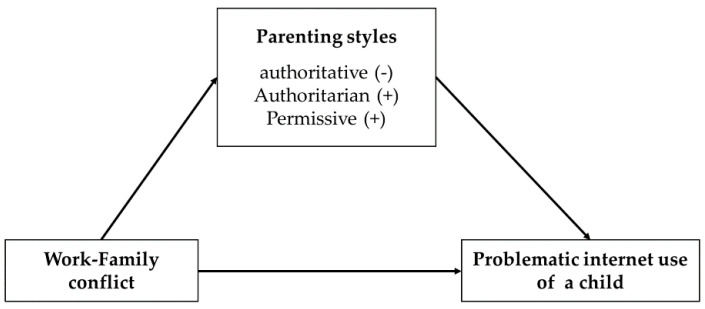
Hypothetical model of mediating of parenting styles on the association between WFC and the PIU of a child.

According to a systematic literature review, individual-level factors such as physiological and psychopathological characteristics are related to the PIU of a child [27]. However, the relationship between parents as environmental factors, and the PIU of a child, has been rarely studied. In particular, the WFC and parenting are the primary concern in society, but little is known about how WFC is related to the PIU of a child. Therefore, this study aims to identify the relationship between maternal WFC and the PIU of a child through the mediating of parenting styles. We hypothesized that maternal WFC is associated with parenting styles, which would in turn be associated with the PIU of a child. We anticipated that parenting style would mediate maternal WFC and the PIU of a child (Figure 1). Based on a previous study, we hypothesized that maternal WFC is positively associated with authoritarian and permissive parenting styles, whilst maternal WFC is negatively associated with authoritative parenting styles.

## 2. Methods

### 2.1. Study Design

This was a cross-sectional correlation study. The study aimed to examine the effect of the association between maternal WFC and the PIU of a child according to parenting styles among 707 working mothers and their children. 

### 2.2. Data and Study Participants

The data were extracted from the 2017 Panel Study on Korean Children (PSKC) by the Korea Institute of Child Care and Education (accessed on 3 September 2020). The PSKC is a nationally representative sample of parents and their children born between April and July 2008. The PSKC dataset is open to the public and freely available for all researchers for academic purposes. The 1st wave was conducted in 2008, and the 10th in 2017. The survey aims to examine the children’s growth and developmental characteristics, parenting, and the effects of childcare supports and policies. The 10th PSKC had data from 2150 families. To clarify our aims, we excluded the data of mothers with disability (689), who were unemployed (712), and incomplete data (42). The final data of the study included 707 working mothers and their children [28]. 

### 2.3. Measurement

#### 2.3.1. Household Income

Household income measurement uses a single question: “What is the average monthly household income for your past year?” We classified the household income based on the lowest 25% of the distribution of household income (≤4 million = 1 vs. > 4 million = 0).

#### 2.3.2. Work–Family Conflict

To assess the WFC, the PSKC adopted the scale of Marshall and Barnett (1993). It has 9 items rated on a 5-point Likert scale ranging from 1 (strongly disagree) to 5 (strongly agree) [29]. WFC is calculated as the average score for each item, and a higher score indicates higher levels of WFC. In the previous study, the Cronbach’s alpha coefficient of the scale was 0.71 [18]. In this study, Cronbach’s alpha was 0.90.

#### 2.3.3. Parenting Styles

Parenting style assessed the parent’s behavior and practices toward their children by the Parenting Styles and Dimensions Questionnaire (PSDQ) [30]. This instrument consists of three typologies: authoritative parenting style (27 questions), authoritarian parenting style (20 questions), and permissive parenting style (15 questions). It includes 62 items rated on a 5-point Likert scale ranging from 1 (very unlikely) to 5 (very likely). Three typologies of parenting style calculated the means of each item. In the previous study, Cronbach’s alpha coefficients of the scale were 0.81 (authoritative parenting style), 0.86 (authoritarian parenting style), and 0.70 (permissive parenting style) [31]. In this study, the Cronbach’s alpha coefficients were 0.91 (authoritative parenting style), 0.87 (authoritarian parenting style), and 0.59 (permissive parenting style).

#### 2.3.4. The PIU of a Child

To assess the PIU of a child, the PSKC adopted the modified version of the Korean internet addiction scale (K-scale) for adolescent observers provided by the Korea Information Society Agency. The modified version adopted the term “PC/smartphone” instead of “Internet”. The “use of PC and smartphone” in the scale means all activities using a media device, such as games, social networking, watching videos, and using the internet. It is 15 items rated on a 4-point Likert scale ranging from 1 (not at all) to 4 (very likely). The scale consists of three domains: disturbance of adaptive functions (5 questions), withdrawal (4 questions), and tolerance (4 questions). The high-risk user group refers to children who score 30 or more, or obtain high scores in all domains (exceeding 14 in disturbance of adaptive functions, 12 in withdrawal, and 11 in tolerance). The potential-risk user group refers to children scoring 28–29 in total, or high scores in at least one domain (exceeding 13 in disturbance of adaptive functions, 11 in withdrawal, or 10 in tolerance). The general user group refers to children scoring 27 in total or less [32]. We coded the high-risk user group and the potential-risk user group into “1” and the other, “0”. In the previous study, the Cronbach’s alpha coefficient of the scale was 0.87 [33]. In this study, Cronbach’s alpha was 0.85.

### 2.4. Data Analysis

All statistical analyses were performed using the SPSS/WIN 23.0 program (IBM, Armonk, NY, USA). In this study, multiple linear or logistic regression analyses were performed depending on whether the dependent variable is binary or continuous. To test the mediating effect of parenting styles in the association between WFC and the PIU, hypothesis testing was performed with three equations examined via multiple linear or logistic regression, according to Baron and Kenny’s method [34].

First, we analyzed and corrected the covariate values related to the PIU of a child (maternal age, educational level of the mother, marital status, subjective health status of the mother, the child’s gender, the subjective health status of the child, internet use time, and self-esteem). Three multiple linear or logistic regression equations were used to test the mediating effect of parenting styles (authoritative, authoritarian, permissive) on the link between WFC and child internet addiction. The PIU of a child was regressed on WFC in the first equation using logistic regression and parenting styles (authoritative, authoritarian, and permissive) and, in the second equation, using multiple linear regression. The PIU of a child was regressed on both WFC and parenting styles in the third equation using logistic regression. Finally, the Sobel test was performed to analyze the mediating effect.

### 2.5. Ethical Consideration

Secondary data from the PSKC were used. These data do not contain respondents’ private information and are available in the public domain. All information was anonymized and de-identified before analysis. Written informed consent was obtained from each participant at the time of recruitment by the Korea Institute of Child Care and Education (KICCEIRB-2018-02).

## 3. Results

### 3.1. General Characteristics of the Participants

The mean maternal age was 40.0 years. A total of 75.2% of mothers had a college degree or higher. In the marital status, divorce, separation, or widowed parents totaled 3.5%. In terms of employment, 63.8% were permanent workers and 11.0% were temporary workers, 20.8% were self-employed workers, and 4.4% were unpaid family workers. A total of 47.2% of mothers rated their health as good. The mean age of the children was 9.4 years, and 48.5% were women. A total of 29.7% of children used the internet for more than 2 h per day. 

Families with a monthly average household income of 4 million won or less were 31.4%. The average of WFC was 2.4 points. The average scores of authoritative, authoritarian, and permissive parenting styles were 3.8, 2.4, and 2.3 points, respectively. The high-risk user group of the PIU among children was 2.5%, and the potential-risk user group was 10.5% (Table 1).

### 3.2. Crude Logistic Regression Relevant to the PIU of a Child

The PIU of a child was associated with the lower maternal educational level (β = −0.62, *p* = 0.009), in addition to marital status, such as divorced/separated/widowed (β = −1.01, *p* = 0.029) (Table 2). Boys had a greater tendency for PIU than girls (β = 0.76, *p* = 0.001). The PIU of a child was associated with poor self-rated health (β = −0.61, *p* = 0.045), prolonged internet use of more than 2 h a day (β = 1.06, *p* < 0.001), and lower levels of self-esteem (β = −0.67, *p* = 0.006).

### 3.3. Mediator Effect of Parenting Styles in the Association of WFC with the PIU of A Child 

WFC was positively associated with the PIU of a child in the first equation (AOR [95% CI] = 1.59 [1.14–2.22], *p* = 0.006) (Table 3). 

In the second equation, WFC was associated with parenting styles. Specifically, WFC was negatively associated with authoritative parenting style (β = −0.06, *p* = 0.002). WFC was positively associated with authoritarian (β = 0.08, *p* = 0.001) and permissive (β = 0.07, *p* < 0.001) parenting styles.

**Figure 2:**
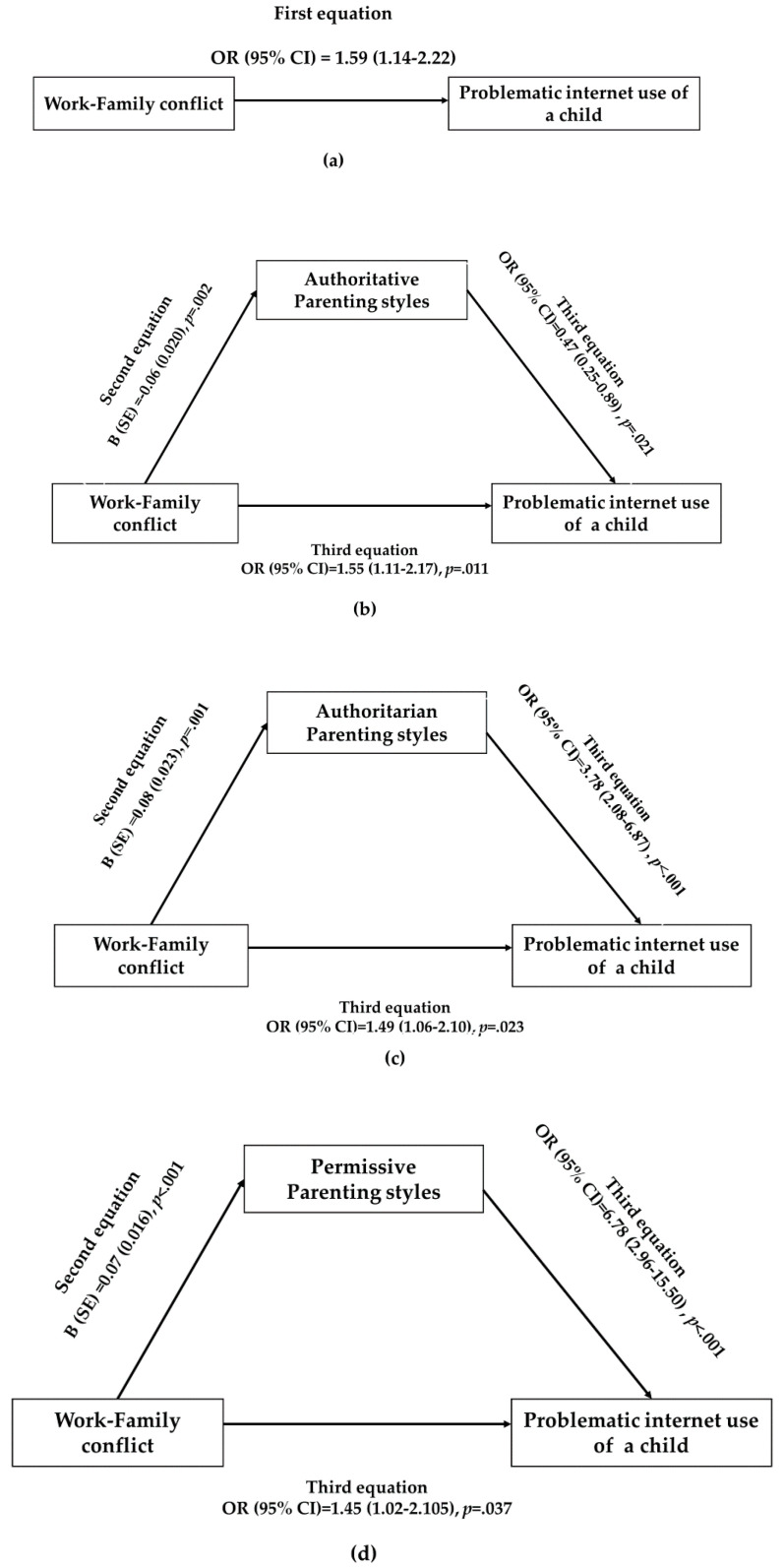
Testing for a mediating effect of parenting styles on the association between WFC and the PIU of a child using logistic regression analysis; (**a**) Direct pathway from WFC to the PIU of a child; (**b**) Indirect pathway from WFC to the PIU of a child via authoritative parenting style; (**c**) Indirect pathway from WFC to the PIU of a child via authoritarian parenting style (**d**) Indirect pathway via permissive parenting style.

In the third equation, parenting styles were linked with the PIU of a child after adjusting for WFC. Furthermore, WFC continued to exert a direct effect on the PIU of a child even though the association was slightly eliminated after adjusting for parenting styles in the third equation rather than the first (authoritative parenting style: AOR [95% CI] = 1.55 [1.11–2.17], *p* = 0.011; authoritarian parenting style: AOR [95% CI] = 1.49 [1.06–2.10], *p* = 0.023; permissive parenting style: AOR [95% CI] = 1.45 [1.02–2.05], *p* = 0.037). The mediating effect of parenting styles was significant in Sobel’s test (authoritative parenting style: z = 2.08, *p* = *0*.037; authoritarian parenting style: z = 2.71, *p* = *0*.007; permissive parenting style: z = 3.14, *p* = 0.002) (Figure 2).

## 4. Discussion

This study confirmed the link between WFC and the PIU of a child through the mediating effect of parenting styles among Korean working mothers.

First, we found the association of the maternal WFC with the PIU of a child. When mother and child interact with each other within the family system, the stresses can be shared as a common experience in the context of emotional and social ecology [35]. From the perspective of spillover and crossover models in the family system, the mother’s WFC can influence the child’s problematic behavior. Working mothers tend to be stressed due to meeting the incompatible demands of work and family. Meanwhile, children reported to be tired and stressed about working mothers [36]. According to Lam (2014), problematic behaviors such as internet addiction are one of the ways to relieve stress or tension [26]. These results show that the negative emotions caused by the mother’s WFC are transferred to the child’s emotions within the family system, and this can appear as problematic behavior of children. Venkatesh et al. [37] reported that higher WFC was associated with the PIU of a child through the mediating effect of parents’ occupational exhaustion. This finding suggests that the occupational fatigue of a mother, mediated by WFC, may be associated with the PIU of a child.

Second, we found the link between WFC and parenting styles. Specifically, higher WFC levels showed a negative association with the authoritative parenting style and a positive association with the authoritarian or permissive parenting style. These findings are consistent with the result of Matejević and Đorđević [21]. Maternal WFC might hurt the quality of parenting within the family system due to occupational exhaustion. Occupational exhaustion, psychological burden, and the tension of the mother caused by role conflict may spill over to the child to show poor parenting behaviors in the family. A working mother who struggles with higher WFC tends to show lower warmth and lower consistency in parenting behaviors [18]. Therefore, to improve parenting quality, strategies and supports for reducing the WFC of working mothers would be essential.

Third, we found the association of the maternal parenting style with the PIU of a child. The authoritative parenting style, characterized as high levels of warmth and consistency in parenting behaviors, showed a negative connection with the PIU of a child. Meanwhile, the authoritarian or permissive parenting styles had a negative association with the PIU of a child. Similarly, according to a study by Hess and Pollmann-Schult (2019), a child’s problematic behavior has a negative association with the authoritative parenting style, while positive associations with the authoritarian or permissive parenting styles [38]. The parent–child relationship plays a vital role in forming a child’s healthy lifestyle in the family system. In this context, many studies have reported the associations of low-quality parent–child relationships with children’s PIU [39]. In the parent–child relationship, children are prone to be more affected by parental warmth and responsiveness than adolescents [40]. Moreover, a mother’s parenting style influences the development and behaviors of a child more than fathers. Mothers tend to play a role in creating a healthy environment in the family system. Maternal guidelines and rules for children’s internet use are necessary. However, excessive restrictions or lack of support for children can lead to a child’s PIU. Therefore, parents should avoid authoritarian and permissive parenting styles that coercively control or neglect children’s internet use. On the other hand, it is necessary to improve parenting behavior with an authoritative parenting style that monitors and supervises internet use by setting reasonable rules in a child’s emotions.

According to a systematic review by Vondráčková and Gabrhelik [27], severe conflicts between parent–child relationships often result in the PIU of a child. Moreover, they insisted that family-based counseling is essential for treating the addiction problem of a child. Parenting experts have also emphasized the importance of quality parenting behavior by improving media use consequences on health in a family-based approach [41]. Sanders et al. [42] provided a triple parenting program with 12 episodes for parents with children. As a result, the children’s media-related problems decreased, and parental confidence in media-related parenting increased. Furthermore, they found maintenance effects on a 6-month follow-up test. Therefore, when planning interventions for parents to improve a child’s PIU, it is necessary to consider the following: explain, plan, and praise children’s desirable media behaviors, help them cope with difficulties, and encourage them to participate in more physical activities.

Finally, we found the link between maternal WFC with the PIU of a child through the mediating effect of parenting style. A previous study reported that high maternal WFC levels cause emotional problems and problematic behaviors, such as hyperactivity, in children by mediating abusive parenting practices [38]. In this context, our study results indicated that there is likely to be poor parenting when mothers experience high levels of WFC. Ultimately, these results seem to lead to children’s PIU.

Despite the significance of the research results, this study has some limitations. Children’s behavior is developed and formed through a parent–child relationship in the family system. Our research focused on the influence of maternal WFC and parenting style on the PIU of a child. In the traditional Korean family system, where women are expected to devote themselves to childrearing and housework, most of the parenting is done primarily by mothers. Our research results can be meaningful in this context. However, further studies including the effects of the father in the family system would be necessary. Furthermore, residual confounding is possible because of excluded variables about the media available in the environment.

## 5. Conclusions

The high WFC of a mother, a primary caregiver, can affect parenting style and consequently cause problematic internet use of children within the family system. Therefore, when planning an intervention to reduce children’s PIU for working mothers, a multifaceted approach is essential, including both WFC and parenting behavior.

## Figures and Tables

**Table 1:** Participant’s general characteristics (N = 707).

Variables	N (%)	Mean ± SD	Range
Mothers			
Age (year)		40.0 ± 3.58	29.0–55.0
Education (≥some college)	532 (75.2)		
Marital status			
Married	682 (96.5)		
Divorced/separated/widowed	25 (3.5)		
Type of employment			
Permanent position	451 (63.8)		
Temporary position	78 (11.0)		
Self-employed	147 (20.8)		
Unpaid family worker	31 (4.4)		
Good self-rated health	334 (47.2)		
Children			
Age		9.4 ± 0.12	9.0–10.0
Sex			
Men	364 (51.5)		
Women	343 (48.5)		
Good self-rated health	628 (88.8)		
Hours of internet use (hours/day)			
≥2 h/day	210 (29.7)		
<2 h/day	497 (70.3)		
Self-esteem		3.5 ± 0.42	1.6–4.0
Household income (million, won)		581.2 ± 441.58	100–7000
Low SES (≤400 million, won)	222 (31.4)		
Work–Family conflict		2.4 ± 0.71	1.0–5.0
Parenting behaviors			
Authoritative parenting style		3.8 ± 0.39	2.3–5.0
Authoritarian parenting style		2.4 ± 0.44	1.2–3.9
Permissive parenting style		2.3 ± 0.31	1.4–3.4
Problematic internet use		24.1 ± 6.25	15.0–55.0
High-risk group	18 (2.5)		
Potential-risk group	74 (10.5)		
General user group	615 (87.5)		

SES = socioeconomic status.

**Table 2:** Unadjusted associations of problematic internet use (N = 707).

Variables	β (SE)	OR (95% CI)	*p*-Value
Mothers			
Age (year)	−0.04 (0.032)	0.96 (0.91–1.03)	0.264
Education (≥some college)	−0.62 (0.238)	0.54 (0.34–0.86)	0.009
Marital status (married)	−1.01 (0.460)	0.37 (0.15–0.90)	0.029
Type of employment (permanent)	0.01 (0.117)	1.01 (0.80–1.27)	0.954
Good self-rated health	−0.88 (0.243)	0.42 (0.26–0.67)	<0.001
Children			
Age (year)	0.25 (0.957)	1.29 (0.20–8.40)	0.791
Gender (men)	0.76 (0.237)	2.14 (1.38–3.41)	0.001
Good self-rated health	−0.61 (0.306)	0.54 (0.30–0.99)	0.045
Hours of internet use (≥2 h/day)	1.06 (0.228)	2.90 (1.85–4.53)	<0.001
Self-esteem	−0.67 (0.244)	0.51 (0.32–0.83)	0.006
Family economic status			
Low SES (≤400 million, won)	−0.23 (0.250)	0.79 (0.49–1.29)	0.350
Work–Family conflict	0.52 (0.156)	1.68 (1.24–2.28)	0.001
Parenting Styles			
Authoritative parenting style	−1.25 (0.299)	0.29 (0.16–0.52)	<0.001
Authoritarian parenting style	1.43 (0.273)	4.16 (2.44–7.11)	<0.001
Permissive parenting style	2.33 (0.403)	10.29 (4.67–22.68)	<0.001

β = unstandardized regression coefficient; CI = confidence interval; OR = odds ratio; SE = standard error; SES = socioeconomic status.

**Table 3:** Hypothesis testing for a mediating effect of parenting style on the association between WFC and the PIU of a child.

Variables	β (SE)	AOR (95% CI)	*p*-Value
Parenting behaviors: Authoritative parenting style			
First equation			
Outcome variable: The PIU of a child			
Independent variable: WFC	0.47 (0.170)	1.59 (1.14–2.22)	0.006
Second equation			
Outcome variable: Authoritative parenting style			
Independent variable: WFC	−0.06 (0.020)		0.002
Third equation			
Outcome variable: The PIU of a child			
Mediator: Authoritative parenting style	−0.76 (0.328)	0.47 (0.25–0.89)	0.021
Independent variable: WFC	0.44 (0.172)	1.55 (1.11–2.17)	0.011
Sobel’s test, z = 2.08, *p* = 0.037			
Parenting behaviors: Authoritarian parenting style			
First equation			
Outcome variable: The PIU of a child			
Independent variable: WFC	0.47 (0.170)	1.59 (1.14–2.22)	0.006
Second equation			
Outcome variable: Authoritarian parenting style			
Independent variable: WFC	0.08 (0.023)	-	0.001
Third equation			
Outcome variable: The PIU of a child			
Mediator: Authoritarian parenting style	1.33 (0.305)	3.78 (2.08–6.87)	<0.001
Independent variable: WFC	0.40 (0.176)	1.49 (1.06–2.10)	0.023
Sobel’s test, z = 2.71, *p* = 0.007			
Parenting behaviors: Permissive parenting style			
First equation			
Outcome variable: The PIU of a child			
Independent variable: WFC	0.47 (0.170)	1.59 (1.14–2.22)	0.006
Second equation			
Outcome variable: Permissive parenting style			
Independent variable: WFC	0.07 (0.016)		<0.001
Third equation			
Outcome variable: The PIU of a child			
Mediator: Permissive parenting style	1.91 (0.422)	6.78 (2.96–15.50)	<0.001
Independent variable: WFC	0.37 (0.178)	1.45 (1.02–2.05)	0.037
Sobel’s test, z = 3.14, *p* = 0.002			

β = unstandardized regression coefficient; AOR = adjusted odds ratio; CI = confidence interval; SE = standard error; WFC = work–family conflict; PIU = problematic internet use. All multiple regression models were adjusted for maternal age, education level, marital status, self-rated health, and children’s sex, self-rated health, hours of internet use, and self-esteem.

## Data Availability

Publicly available datasets were analyzed in this study. These data can be found here: https://panel.kicce.re.kr/panel/module/rawDataManage/index.do?menu_idx=56 (accessed on: 3 September 2020).
